# Factors influencing the SARS-CoV-2 infection and vaccination induced immune response in rheumatoid arthritis

**DOI:** 10.3389/fimmu.2022.960001

**Published:** 2022-10-12

**Authors:** Dora Nemeth, Hajnalka Vago, Laszlo Tothfalusi, Zsuzsanna Ulakcsai, David Becker, Zsofia Szabo, Bernadett Rojkovich, Bela Merkely, Gyorgy Nagy

**Affiliations:** ^1^ Department of Rheumatology and Clinical Immunology, Semmelweis University, Budapest, Hungary; ^2^ Department of Internal Medicine and Oncology, Semmelweis University, Budapest, Hungary; ^3^ Department of Genetics, Cell- and Immunobiology, Semmelweis University, Budapest, Hungary; ^4^ Heart and Vascular Center, Semmelweis University, Budapest, Hungary; ^5^ Department of Sports Medicine, Semmelweis University, Budapest, Hungary; ^6^ Department of Pharmacodynamics, Semmelweis University, Budapest, Hungary; ^7^ Department of Laboratory Medicine, Semmelweis University, Budapest, Hungary; ^8^ Buda Hospital of the Hospitaller Order of Saint John of God, Budapest, Hungary

**Keywords:** COVID-19, rheumatoid arthritis, antibody response, T-cell response, vaccination, DMARD (disease-modifying antirheumatic drug treatment), targeted therapy

## Abstract

**Background:**

To investigate the factors that have significant impact on the Severe Acute Respiratory Syndrome Corona Virus 2 (SARS-CoV-2) infection and vaccination induced immune response in rheumatoid arthritis (RA).

**Methods:**

Serological response was measured by quantifying anti-SARS-CoV-2 specific antibodies, while the cell-mediated response was measured by a whole-blood test quantifying the interferon (IFN)-γ response to different SARS-CoV-2-specific domains.

**Results:**

We prospectively enrolled 109 RA patients and 43 healthy controls. The median time (IQR) between the confirmed infection or the last vaccination dose and the day when samples were taken (“sampling interval”) was 3.67 (2.03, 5.50) months in the RA group. Anti-Spike (anti-S) specific antibodies were detected in 94% of RA patients. Among the investigated patient related variables, age (p<0.004), sampling interval (p<0.001), the brand of the vaccine (p<0.001) and targeted RA therapy (TNF-inhibitor, IL-6 inhibitor, anti-CD20 therapy) had significant effect on the anti-S levels. After covariate adjustment TNF-inhibitor therapy decreased the anti-S antibody concentrations by 80% (p<0.001). The same figures for IL-6 inhibitor and anti-CD20 therapy were 74% (p=0.049) and 97% (p=0.002), respectively. Compared to subjects who were infected but were not vaccinated, the RNA COVID-19 vaccines increased the anti-S antibody levels to 71.1 (mRNA-1273) and 36.0 (BNT162b2) fold (p<0.001). The corresponding figure for the ChAdOx1s vaccine is 18.1(p=0.037). Anti-CCP (anti-cyclic citrullinated peptides) positive patients had 6.28 times (p= 0.00165) higher anti-S levels, than the anti-CCP negative patients. Positive T-cell response was observed in 87% of the healthy volunteer group and in 52% of the RA patient group. Following vaccination or infection it declined significantly (p= 0.044) but more slowly than that of anti-S titer (6%/month versus 25%). Specific T-cell responses were decreased by 65% in patients treated with anti-CD20 therapy (p=0.055).

**Conclusion:**

Our study showed that the SARS-CoV-2-specific antibody levels were substantially reduced in RA patients treated with TNF-α-inhibitors (N=51) and IL-6-inhibitor (N=15). In addition, anti-CD20 therapy (N=4) inhibited both SARS-CoV-2-induced humoral and cellular immune responses. Furthermore, the magnitude of humoral and cellular immune response was dependent on the age and decreased over time. The RNA vaccines and ChAdOx1s vaccine effectively increased the level of anti-S antibodies.

## Introduction

The COVID-19 pandemic caused by the SARS-CoV-2 has appeared as a new disease with a serious global health effect, challenging clinical management (1–3).

Patients with autoimmune inflammatory rheumatic diseases have been considered clinically vulnerable to SARS-CoV-2 infection. These patients were shown to be at an increased risk of hospitalization and death from SARS-CoV-2 (COVID) infection than the general population, and they have been considered a priority target group for vaccine administration (4, 5).

As a prevention of severe disease, numerous vaccines have been developed in the general population (6–9). However, immunogenicity and clinical effectiveness in patients receiving immunosuppression who are at risk of diminished immune response are not clarified.

Here we investigated the COVID-specific antibody and T-cell responses in RA patients treated with various immunosuppressive therapies.

## Materials and methods

### Patients

All RA patients were recruited in the rheumatology outpatient department of Semmelweis University (Buda Hospital of the Hospitaller Order of Saint John of God, Budapest, Hungary) (approval number IV/2021-1/2021/EKU) from 12.08.2021 until 14.12.2021. Patients ≥ 18 years of age, were vaccinated against COVID-19 and/or had previous SARS-CoV-2 infection and diagnosed with RA (N=109) according to the 2010 American College of Rheumatology/European League Against Rheumatism classification criteria (10) were enrolled. The demographic data and the clinical parameters of the patients are summarized in **Table 1**. The treatments of RA patients were explained in **Table 2**.** A** control group of 43 healthy individuals was included who were vaccinated against COVID-19 and/or had previous SARS-CoV-2 infection. Written, informed consent was obtained from all participants.

**Table 1:** Demographics.

Variable	Healthy, N = 43^1^	RA, N = 109^1^	p-value^2^
Gender			<0.001
Male	25 (58%)	14 (13%)	
Female	18 (42%)	95 (87%)	
Age	35 (29,48)	59 (46,66)	<0.001
Vaccination			<0.001
ChAdOx1s	1 (2.3%)	5 (4.6%)	
BNT162b2	34 (79%)	75 (69%)	
Gam-COVID	4 (9.3%)	1 (0.9%)	
mRNA-1273	0 (0%)	19 (17%)	
BBIBP-CorV	0 (0%)	5 (4.6%)	
Sampling Interval (months)	3.80 (1.73, 6.85)	3.67 (2.03, 5.50)	0.3
COVID status			0.13
Recovered from COVID-19 (unvaccinated)	4 (9.3%)	4 (3.7%)	
Recovered from COVID-19 and vaccinated	16 (37%)	30 (28%)	
Vaccinated (no COVID infection)	23 (53%)	75 (69%)	
Positive anti-S response (N)	36 (100%)	102 (94%)	
Positive T-cell response (CD4^+^ or CD8^+^) (N)	34 (87%)	57 (52%)	
anti-S response (U/ml)	1,708 (686, 6,579)	724 (99, 3,909)	0.017
COVID spike-specific CD4^+^ T-cell response (IU/ml)	0.41 (0.16, 1.07)	0.08 (0.02, 0.41)	<0.001
COVID spike-specific CD4^+^ and CD8^+^ T-cell response (IU/ml)	0.53 (0.30, 1.32)	0.10 (0.03, 0.46)	<0.001
Whole COVID virus-specific CD4^+^ and CD8^+^ T-cell response (IU/ml)	0.60 (0.30, 1.56)	0.16 (0.04, 0.94)	<0.001

^1^n (%); Median (IQR).

^2^Pearson’s Chi-squared test; Wilcoxon rank sum test; Fisher’s exact test.

The “sampling interval” is the time interval in months (1 month = 30 days) between COVID infection or the last vaccine administration and laboratory sampling.

**Table 2:** Treatments by drug categories.

Characteristic	N = 109^1^
Targeted therapies	
anti-CD20	4 (3.7%)
IL-6 inhibitor	15 (14%)
JAK inhibitor	5 (4.6%)
TNF-α inhibitor	51 (47%)
MTX	64 (59%)
csDMARDs other than MTX	16 (15%)
CCS	25 (23%)

^1^n (%).

csDMARDs, conventional synthetic disease-modifying antirheumatic drugs; CCS, corticosteroids.

### Measurement of SARS-CoV-2 specific antibodies (Elecsys^®^ Anti-SARS-CoV-2 S assay, Roche)

SARS-CoV-2 specific antibodies were analysed using Elecsys Anti-SARS-CoV-2 S immunoassay (Roche Diagnostics International Ltd, Switzerland) on Cobas e6000 instrument. The test detects** **antibodies specific to SARS-CoV-2 spike (S) protein receptor binding domain (RBD) in human serum and plasma. The assay uses a recombinant protein representing the RBD of the S antigen in a double-antigen sandwich assay format, which favors detection of high affinity antibodies (IgG, IgA, IgM) against SARS-CoV-2 in a quantitative manner. The method is based on double-antigen sandwich principle using electrochemiluminescence for quantitative determination of antibodies. The limit of quantification is 0.4 U/ml. Results below 0.8 U/ml are considered negative and results equal to or** **above 0.8 are considered positive due to the test description (11).

### Determination of IFN-γ T-cell responses (QuantiFERON (QIAGEN Group))

1 ml of heparinized whole blood was stimulated with specific antigens (S1, S2, RBD subdomains), eliciting CD4^+^ (Ag1) and CD4^+^-CD8^+^ (Ag2) T-cells immune responses. One tube contained additional specific peptides, covering the S (spike), N (nucleocapsid) and M (M protein) domains, and all other domains of the SARS-CoV-2 to elicit a more complete specific (Ag3) T cell-mediated immune response. After 16-24 hours of incubation at 37°C, IFN-γ levels were measured by an enzyme-linked immunosorbent assay (ELISA QuantiFERON (QIAGEN Group)), following the manufacturer’s instructions. Based on the data sheet provided by the manufacturer, early data suggested an IFN-γ cut-off for positivity between 0.15 IU/ml and 0.2 IU/ml (12). In our study the positive cut-off was 0.15 IU/ml.

### Statistical analysis

Demographic and immunogenicity variables were summarized by using descriptive statistics. Categorical variables were reported as count and proportion, while continuous variables, including whole COVID virus-specific CD4+ and CD8+ T-cell response and anti-S antibodies were reported as median and interquartile range (IQR). Statistical imbalances across the treatment groups (healthy versus RA patients) were calculated with nonparametric tests. The variable “Sampling Interval” was defined as the time interval (in months) between the vaccination or COVID infection and the time when blood samples were taken for the laboratory analysis. For numerical convenience, we reported the effect of this variable in months (one month consists of 30 days), but in the actual calculations we used the real dates, i.e., days. Age, gender, sampling interval, differences between vaccines’ efficiencies were considered as confounding variables. The response variables were anti-S antibodies (specific antibodies for the SARS-CoV-2 spike glycoprotein) representing the humoral immunogenicity response and whole COVID virus-specific CD4+ and CD8+ T-cell response. The effect of the following explanatory factor variables were tested on the response variables: cohort (healthy or RA patients), methotrexate treatment (MTX), DMARD (disease-modifying antirheumatic drug treatment) (MTX, leflunomide), steroid treatment and targeted treatment. Regarding the latter, to increase the statistical power of the analysis, the levels of the targeted treatment variable were constructed by lumping drugs with common targets such as TNF antagonists (etanercept, infliximab), CD20 antagonist (rituximab), JAK- kinase inhibitors into one class. An additional “base” category of this variable was set for healthy volunteers and patients who were not getting any targeted treatment. Similarly, the “Vaccine” variable had six levels. For labeling the levels we used the commonly used names in Hungary such as: BNT162b2, mRNA-1273, ChAdOx1s, Gam-COVID, BBIBP-CorV., The additional sixth level (“Infected”) stands for who was infected but never vaccinated. We used linear regression with logarithmically transformed response variables to eliminate the effects of confounding variables. The initial regression model included the following variables: “RA patient” (reference to the two cohorts), Age (years), Gender (male/female), Sampling interval (months), Targeted therapy (six levels), Vaccine (also six levels) MTX (yes/no) and Steroid (yes/no). This initial model was reduced by selecting the best subset of predictors the using the R program package leaps (13). We reported the exponentiated regression parameters which correspond to geometric mean ratios regarding to the baseline category or to one unit change for the continuous explanatory variables. The significance of the exploratory variable on the response variables was judged by significance of the regression parameters; no correction for multiple hypothesis testing was made. Data were analyzed using R (14), tables and figures were prepared with the gtsummary (15) and ggplot2 (16).

## Results

### Study population

109 RA patients and 43 healthy controls were included in the study (**Table 1** **).** RA disease activity was evaluated by clinical examination through the disease activity score 28-CRP (DAS28-CRP), DAS28-CRP median was 2.6. A DAS28-CRP value greater than 5.1 indicates high disease activity, >3.2 to ≤5.1 means moderate, 2.6 to ≤3.2 means low disease activity and 0 to <2.6 indicates remission (17). In our study we analyzed the data of the basic schedule of 2 vaccines in all participants. The majority of RA patients and controls received the BNT162b2 vaccine (N=75 and N=34, respectively), 19 participants received mRNA-1273 vaccine. Participants vaccinated with modified adenovirus technology such as ChAdOx1s vaccine (N=6) or Gam-COVID vaccine (N=5) were also included. Furthermore, 5 participants were vaccinated with BBIBP-CorV vaccine. The median interval (IQR) between the first day of confirmed COVID infection or the day of last vaccination was 3.80 (1.73, 6.85) months for the control group and 3.67 (2.03, 5.50) months for the RA group respectively. A total of 75 RA patients (69%) and 23 controls (53%) were vaccinated against COVID-19 (no previous SARS-CoV-2 infection); 30 RA patients (28%) and 16 controls (37%) were vaccinated against COVID-19 and had previous SARS-CoV-2 infection (**Table 1**). 4 RA patients (3.7%) and 4 controls (9.3%) were unvaccinated and had previous SARS-CoV-2 infection (**Table 1**). 33 RA patients had COVID infection, all of them had symptoms associated with COVID. Significant differences were found with respect to age (p<0.001) and gender (p<0.001) between the two groups (**Table 1** **).** The majority of RA patients were treated with csDMARD (conventional synthetic disease-modifying antirheumatic drug - MTX, leflunomide, sulphasalazine, hydroxychloroquine) (N=80, 73%) with or without low dose of corticosteroids (CCS) (below 7.5 mg/day prednisone or equivalent), most of them with MTX (N=64, 59%). 25 patients (23%) received low dose of corticosteroid therapy. In the RA group 51 (47%) patients were treated with TNF-α inhibitors (adalimumab, etanercept, certolizumab, infliximab or golimumab), 15 (14%) with IL-6-inhibitor (tocilizumab), 4 (3.7%), with rituximab therapy and 5 (4.6%), and patients were treated with JAK inhibitors (baricitinib, tofacitinib; in all cases with or without DMARD/CCS, **Table 2**). For the assessment of the healthy COVID-specific immune responses, 36 participants provided sample for serological analysis and 39 for the analysis of T-cell specific responses.

### Anti-SARS-CoV-2 antibody responses

**Figure 1:**
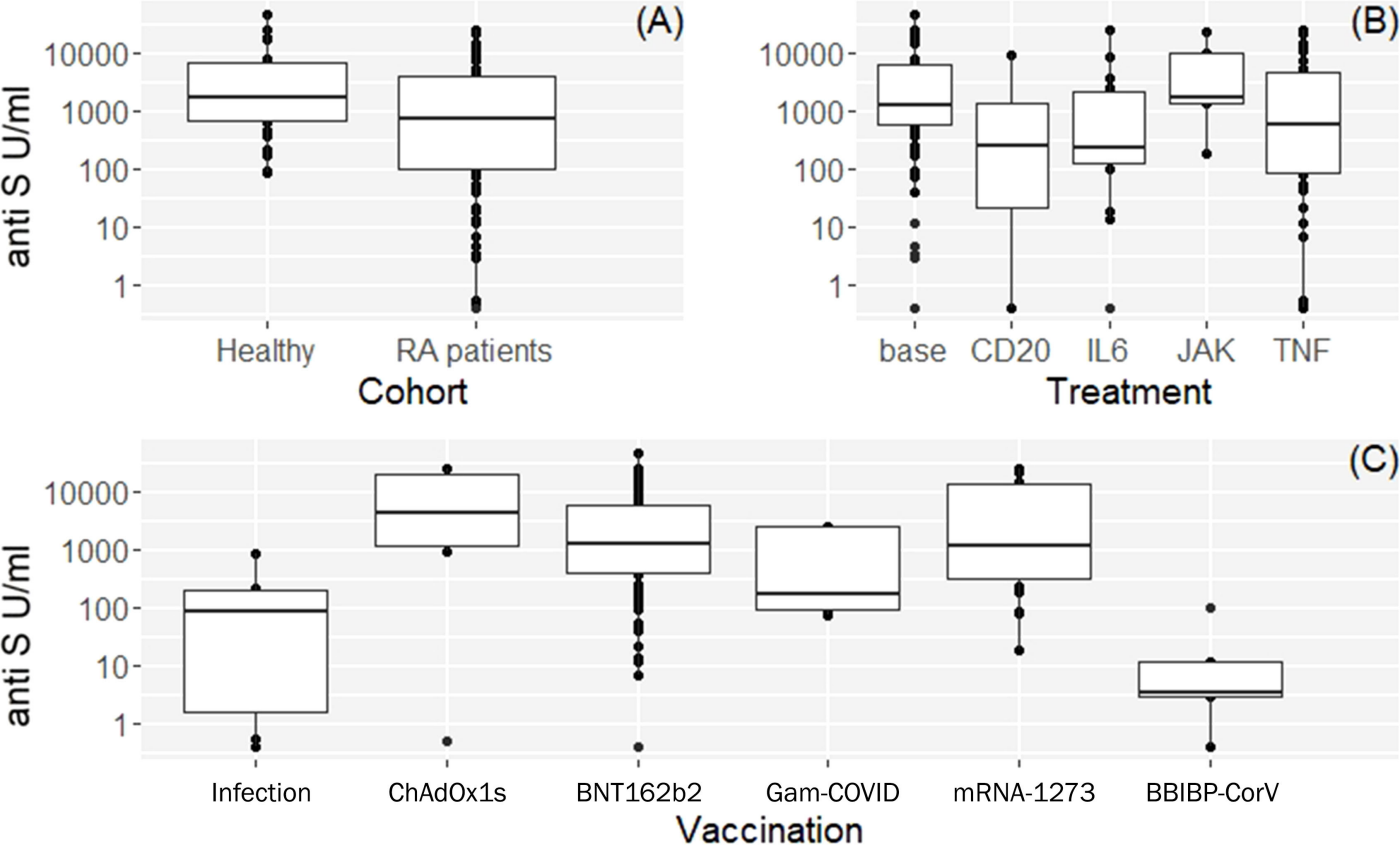
Box plots of vaccine or infection induced anti-S antibody levels and demographic characteristics of the study participants. **(A)** Observed anti-S antibody levels by cohorts. **(B)** Anti-S antibody levels by targeted drug treatment modalities in the combined data set (both cohorts). Base: means healthy volunteers or RA patients on csDMARDs and/or steroids. **(C)** Anti-S antibody levels after different vaccines available in Hungary in RA patients and healthy volunteers. See **Table 1** for additional and more detailed numeric data.

**Figure 2:**
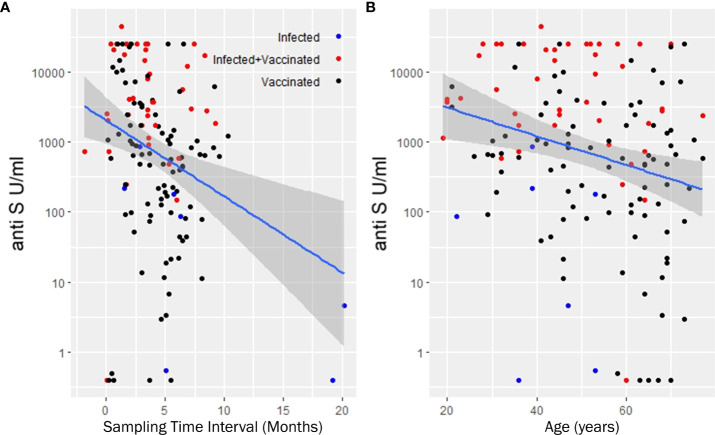
Least squares trend lines of anti-S antibody titers vs time since last vaccination **(A)** and anti-S antibody titers vs. age of recipients **(B)**. **(A, B)** show the antibody titers in the entire study population. The lines are population regression lines unadjusted for any covariates. The shaded bands represent the pointwise 95% confidence interval on the fitted values. We used multiple linear regression to eliminate the covariate effects and get more precise numerical estimate of the slopes. Accordingly, anti-S antibody levels decreases monthly by 25% and each additional year corresponds to 4 percent decrease in the initial anti-S antibody levels. Samples taken from the three subcohort groups (infected, infected but later vaccinated, vaccinated but without record of infection are denoted with different colors).

The seropositivity rate was 94% (102/109) in patients with RA compared with 100% (36/36) in controls (**Table 1**). The median (IQR) concentration of the anti-S antibodies was 724 U/ml (99, 3909) in the RA group (**Table 1** and **Figure 1A** **).** Anti-S antibody levels by targeted drug treatments in the combined data set are shown in **Figure 1B**. **Figure 1C** shows the concentration of COVID-specific antibody after different types of vaccines in the whole study population. In participants who received the mRNA-1273 vaccine, the anti-S antibody levels were 71.1 times (p<0.001) higher than in unvaccinated patients with previous SARS-CoV-2 infection (**Table 3**). Furthermore, BNT162b2 vaccination raised the levels of anti-S antibodies by 36 times (p<0.001) and ChAdOx1s vaccine by 18.1 times (p=0.037), compared to unvaccinated participants (**Table 3**). **Figure 2** shows that subjects who were vaccinated following infection (red dots) had higher anti-S titer compared to those, who had not been infected (black dots), similarly to other observations (18). Based on our optimized regression model (**Table 3**), age and longer sampling interval had a significant impact on the magnitude of the COVID-specific antibody levels in the whole study population. Anti-S antibody levels decreased by 25% (p<0.001), following COVID infection or vaccination in each month (**Figure 2A** **).** Age has also a significant effect, we estimate that every year is associated with a 4% (p=0.004) decrease (**Figure 2B**). The data on the effects of different therapies show that MTX therapy had no significant effect on the anti-S antibody response (N=64; p=0.087) (**Supplementary Table 1**). In addition, the anti-CD20 therapy had an effect on the anti-S antibody titres. In the case of rituximab-treated RA patients, the levels of COVID-specific antibodies were 97% lower than expected compared to patients who were not treated with anti-CD20 (N=4; p=0.002) (**Table 3**). TNF-α inhibitors decreased anti-S antibody levels by 80% (N=51; p<0.001) and IL-6 inhibitor by 74% (N=15; p=0.049) (**Table 3**). By contrast, no significant effect was found in the case of the specific antibody response of patients treated with JAK inhibitors (N=5; p=0.6) (**Table 3**).

**Table 3:** Parameters of the optimized regression model for anti-S antibodies.

Characteristic	exp (Beta)	95% CI^1^	p-value
Sampling Interval	0.75	0.65, 0.86	<0.001
Vaccination			
ChAdOx1s	18.1	1.22, 269	0.037
BNT162b2	36.0	5.51, 235	<0.001
Gam-COVID	3.08	0.19, 49.9	0.4
mRNA-1273	71.1	8.74, 579	<0.001
BBIBP-CorV	0.25	0.02, 4.16	0.3
Age	0.96	0.93, 0.99	0.004
Targeted therapies			
anti-CD20	0.03	0.00, 0.26	0.002
IL-6 inhibitor	0.26	0.07, 0.98	0.049
JAK inhibitor	1.75	0.20, 15.2	0.6
TNF-α inhibitor	0.20	0.08, 0.49	<0.001

In a patient who is treated with a TNF antagonist the expected anti-S antibody level is 80% smaller (p<0.001) and who is treated with a IL-6 inhibitor the anti-S antibody level is 74% lower (p=0.049). The effect of a CD20 antagonist is very pronounced, anti-S antibody level is only 3% of the corresponding control (p=0.002). In each month the anti-S antibody level decreases by 25% (p<0.001). Each additional age corresponds to a 4 percent decrease in anti-S antibodies (p=0.004).

^1^CI, Confidence Interval.

**Figure 3:**
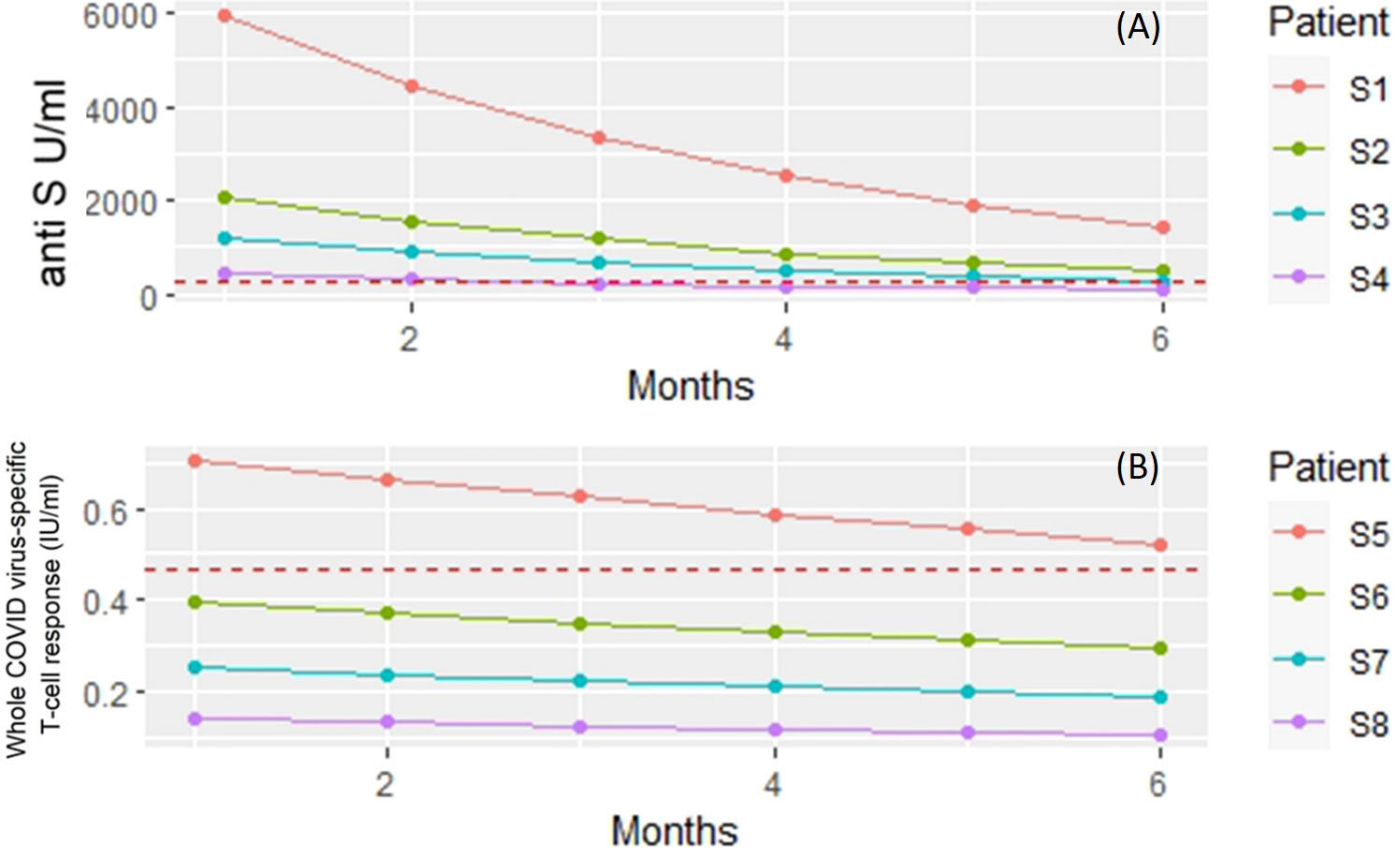
Linear regression models were built to investigate the dependence of anti-S titer and T-cell response on patient and treatment specific factors. The figure shows the model predicted responses for four hypothetical patients (S1 – S8). **(A)** Predicted anti-S antibody concentrations following vaccination with BNT162b2. Subject 1 (S1) - 50 years old, no biology (TNF) treatment. Subject 2 (S2) - 75 years of age but no treatment with TNF inhibitor. Subject 3 (S3) - 50 years old and gets TNF inhibitor. Subject 4 (S4) 75 years of age and receiving TNF inhibitor therapy. Red dashed horizontal line: measured mean anti-S antibody levels in unvaccinated patients and volunteers. **(B)** Predicted whole COVID virus-specific CD4+ and CD8+ T-cell responses following vaccination with BNT162b2. Subject 1 (S5) - 50 years old, no biological (rituximab) treatment. Subject 6 (S6) - 75 years of age but no treatment with rituximab. Subject 7 (S7) - 50 years old and gets rituximab. Subject 8 (S8) 75 years of age and receiving rituximab therapy. Red dashed horizontal line: measured mean whole COVID virus-specific CD4+ and CD8+ T-cell response levels in unvaccinated patients and volunteers.

The covariate adjusted effects of the disease itself were 0.42 (i.e. 58% decrease; p=0.3) for COVID-specific anti-S antibody response. The estimated effect of the immunosuppressant drugs potentially can confound the disease as a contributing factor itself. However, **Supplementary Table 1** shows that the estimated negative effect on the anti-SARS CoV-2 responses remained essentially the same regardless whether the Group factor (control, RA) was included into the regression model or not. Furthermore, these results did not change when the analysis was narrowed down to the patient group (**Supplementary Table 2**). The results of the statistical analysis are illustrated with four hypothetical cases. **Figure 3A** shows the expected anti-S antibody curves of 4 cases (S1-S4) following vaccination with BNT162b2. In the S4 case (75 years old, treated with TNF-α inhibitor) was the lowest expected anti-S antibody curve due to older age and TNF-α inhibitor therapy.

### IFN-γ T-cell responses

**Figure 4:**
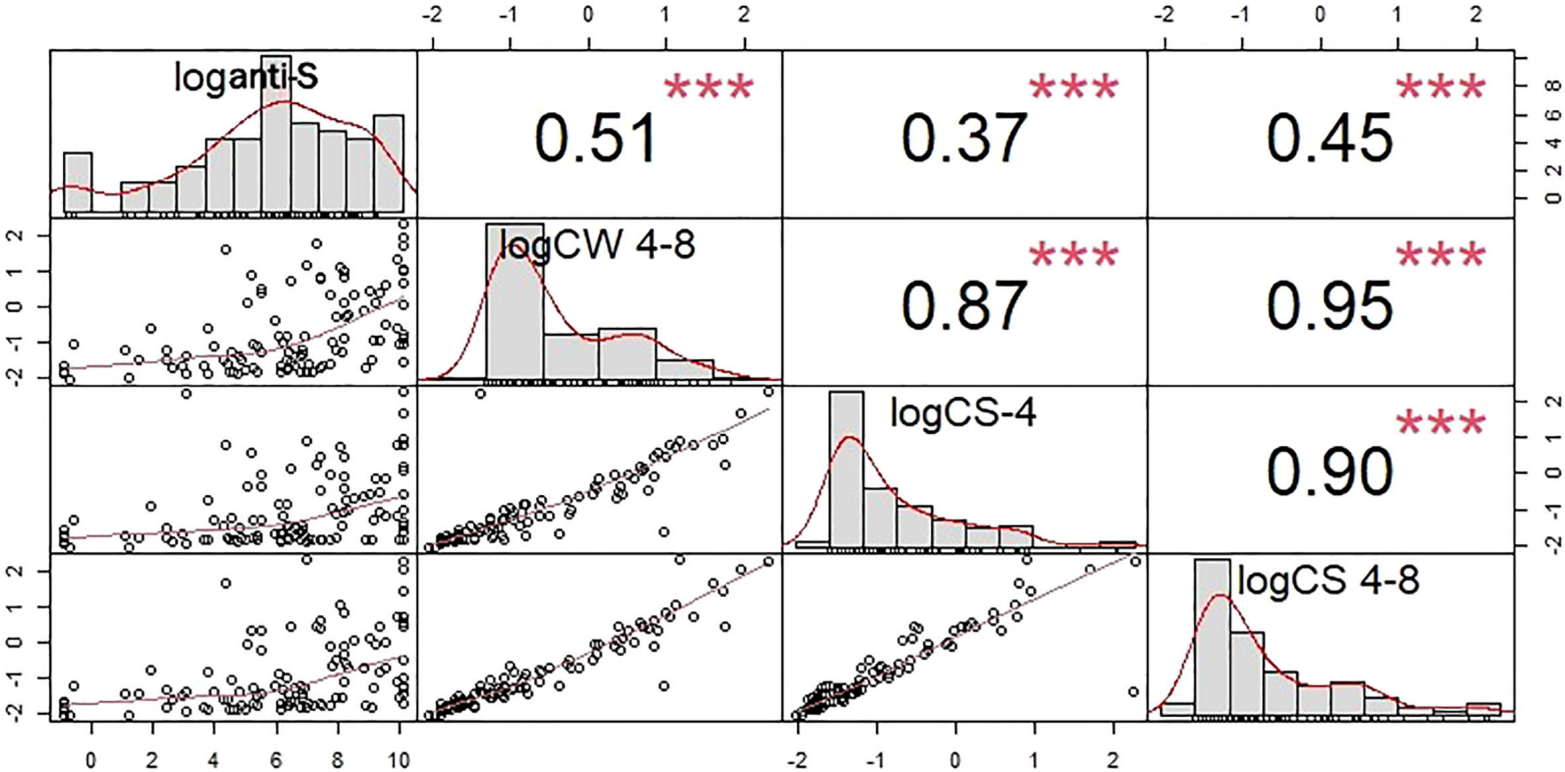
Cross-correlation plots between the humoral and T-cell specific response markers. The square of the correlation coefficients between of humoral and T cell specific immune responses are shown in the upper right triangle. Correlations were computed after the taking the logarithms of the corresponding concentrations. COVID -specific CD4+ activation was assayed by measuring CS-4, CD4+ and CD8+ activation by CS 4-8, and the whole COVID virus-specific CD4+ and CD8+ T-cell response by CW 4-8. loganti-S – logarithm of anti-S antibody concentration*** p<0.001. T-cell specific response markers are strongly correlated with each other. Because of that the whole COVID virus-specific CD4+ and CD8+ T-cell response was in the statistical analysis.

A robust correlation was observed between the anti-S antibody level and the COVID-specific T-cell response (r=0.51, p<0.001) (**Figure 4**). Studying the T cell-mediated immune response following stimulation of the three specific antigens (i: spike antigen stimulating COVID-specific CD4+; ii: spike antigen -stimulating COVID-specific CD4+ and CD8+ and iii: whole COVID virus stimulating COVID specific CD4+ and CD8+ T-cell responses) enabled us to analyze the overall COVID-induced cellular immune response. T-cell specific response markers- COVID spike-specific CD4+, COVID spike-specific CD4+ and CD8+, whole COVID virus-specific CD4+ and CD8+ T-cell response - strongly correlated with each other (r = 0.87, 0.95, 0.9 respectively, p<0.001) (**Figure 4**). Based on these data we analyzed further the whole COVID virus-specific CD4+ and CD8+ T-cell response because the two other COVID-specific T-cell markers (COVID spike-specific CD4^+^ and COVID spike-specific CD4^+^ and CD8^+^) were considered less informative.

57 RA patients (N=57/109; 52%) and 34 controls (N=34/39; 87%) showed a positive COVID-specific T-cell response (**Table 1**). We identified a median IFN-γ level (reflecting whole COVID virus-specific CD4+ and CD8+ T-cell response) of 0.60 IU/ml (0.30, 1.56) in healthy controls; however, the magnitude of the RA patients’ response was 0.16 IU/ml (0.04, 0.94) (**Table 1** **).** In the whole study population we found that the COVID-specific T-cell mediated immune response was age dependent. Older patients had lower response; the estimated decrease was 2%/year (p<0.001) (**Table 4**). The initial immune response decreases gradually. The decrease is slower compared to anti-SARS-CoV-2 antibodies; it is 6% (p=0.044) in each month (**Table 4**). Compared to COVID infection, none of the vaccines had significant effects on COVID-specific T-cell response (**Supplementary Table 3**). Neither TNF-α inhibitors (N=51; p=0.7) nor IL-6 inhibitor (N=15; p>0.9) or JAK inhibitors (N=5; p=0.6) had significant effects (**Table 4**) on COVID specific T-cell response. By contrast, anti-CD20 therapy decreased the COVID-specific T-cell response , on average by 65% (N=4; p=0.055) compared to a patient who had not received anti-CD20 therapy but otherwise all covariates are the same (**Table 4**).

**Table 4:** Parameters of the optimized regression model for whole COVID virus-specific CD4+ and CD8+ T-cell response.

Characteristic	exp (Beta)	95% CI^1^	p-value
Sampling Interval	0.94	0.89, 1.00	0.044
Age	0.98	0.96, 0.99	<0.001
Targeted therapies			
anti-CD20	0.35	0.12, 1.01	0.055
IL-6 inhibitor	0.98	0.53, 1.79	>0.9
JAK inhibitor	1.25	0.48, 3.22	0.6
TNF-α inhibitor	0.94	0.63, 1.39	0.7

Both cohorts. This parameter also shows age dependence (2% decrease by each year) (p<0.001). In each month the whole COVID virus-specific CD4+ and CD8+ T-cell response decreases by 6% (p=0.044). In a patient who is treated with anti-CD20 therapy the expected T-cell response is 65% smaller (p=0.055).

^1^CI, Confidence Interval.

**Figure 3B** shows the expected whole COVID virus-specific CD4+ and CD8+ T-cell response curves of 4 cases (S5-S8) following vaccination with BNT162b2. In the S8 case (75 years old, treated with rituximab) was the lowest expected whole COVID virus-specific CD4+ and CD8+ T-cell response curve due to older age and the rituximab therapy. The Group effect (healthy, RA) was borderline significant (p=0.061) when all available covariates were included into the model (**Supplementary Table 3**) and the results did not change when the analysis was narrowed down to the patient group (**Supplementary Table 4**).

### The effect of CRP, DAS28-CRP, anti-CCP, RF on the COVID-specific immune responses

**Figure 5:**
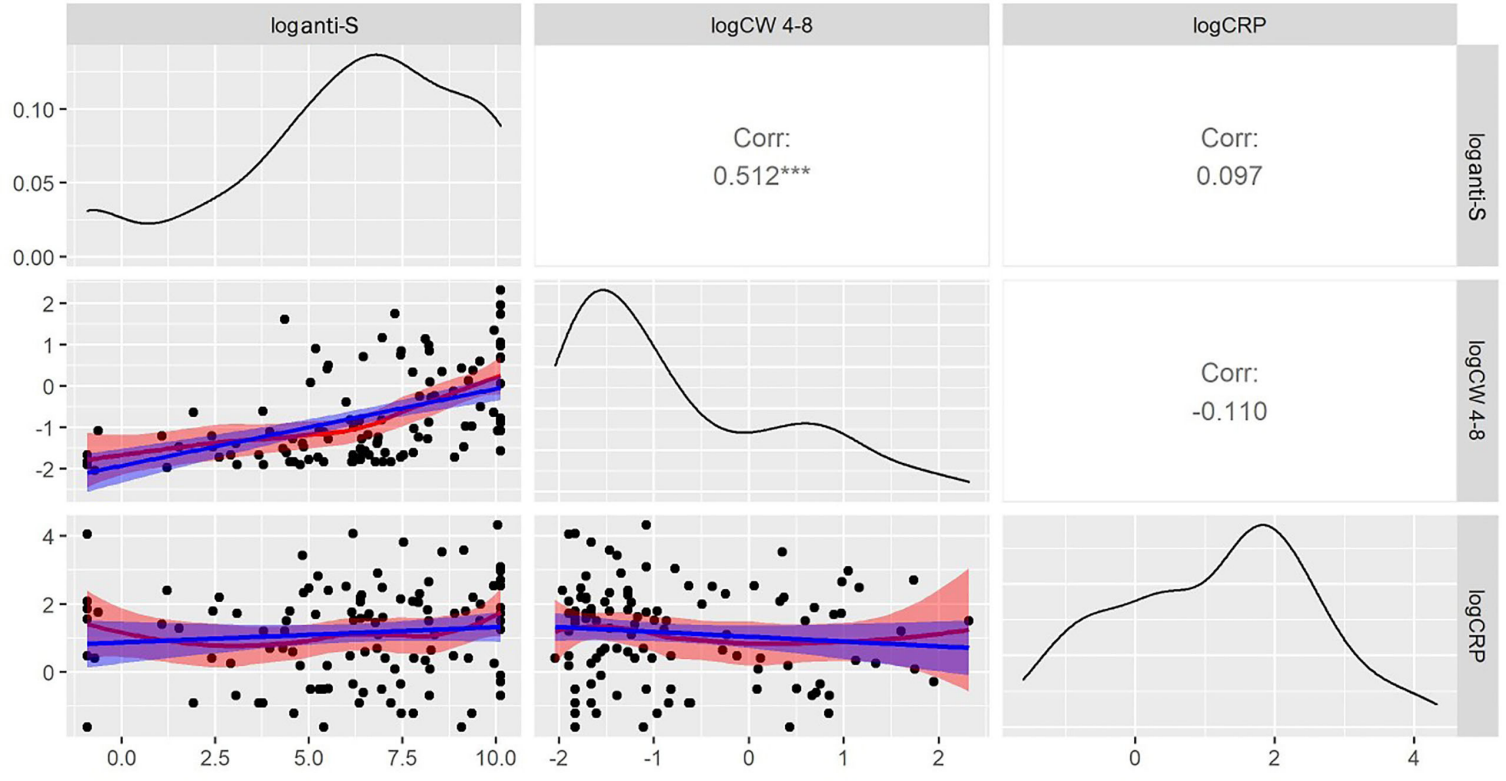
Relationships between CRP and anti-S antibody and whole COVID virus-specific CD4+ and CD8+ T-cell response (CW 4-8) levels. In the lower triangle the variables are plotted against each-other, in the diagonals the smoothed distribution of the variables is shown while the squared correlation coefficients between the variables are shown in the upper right diagram. Log CRP concentrations are correlated neither with CW 4-8 nor with anti-S antibody levels. Because of the lack of correlation, the immune response to vaccines (or infection) seems to be unrelated to the inflammatory condition of RA patients. *** p<0.001.

**Figure 5** shows that there was no correlation between CRP levels and IFN-γ levels (r=-0.110) and anti-S antibody levels (r=0.097). These data suggest that moderate systemic inflammation had no significant effect on the different immune responses against COVID-19. We additionally investigated the connection between the presence of anti-CCP (anti-cyclic citrullinated peptides) antibodies, RF (rheumatoid factor-IgG), DAS28-CRP and the COVID-specific immune responses. Baseline aCCP and RF were considered dichotomously (seropositive (aCCP: ≥20 CU; RF: ≥12 U/ml) vs. seronegative (aCCP: <20 CU; RF: <12 U/ml)) and first we tested the effects of these variables adding individually them to the established regression model. Data of healthy volunteers were excluded from the analysis.

**Figure 6:**
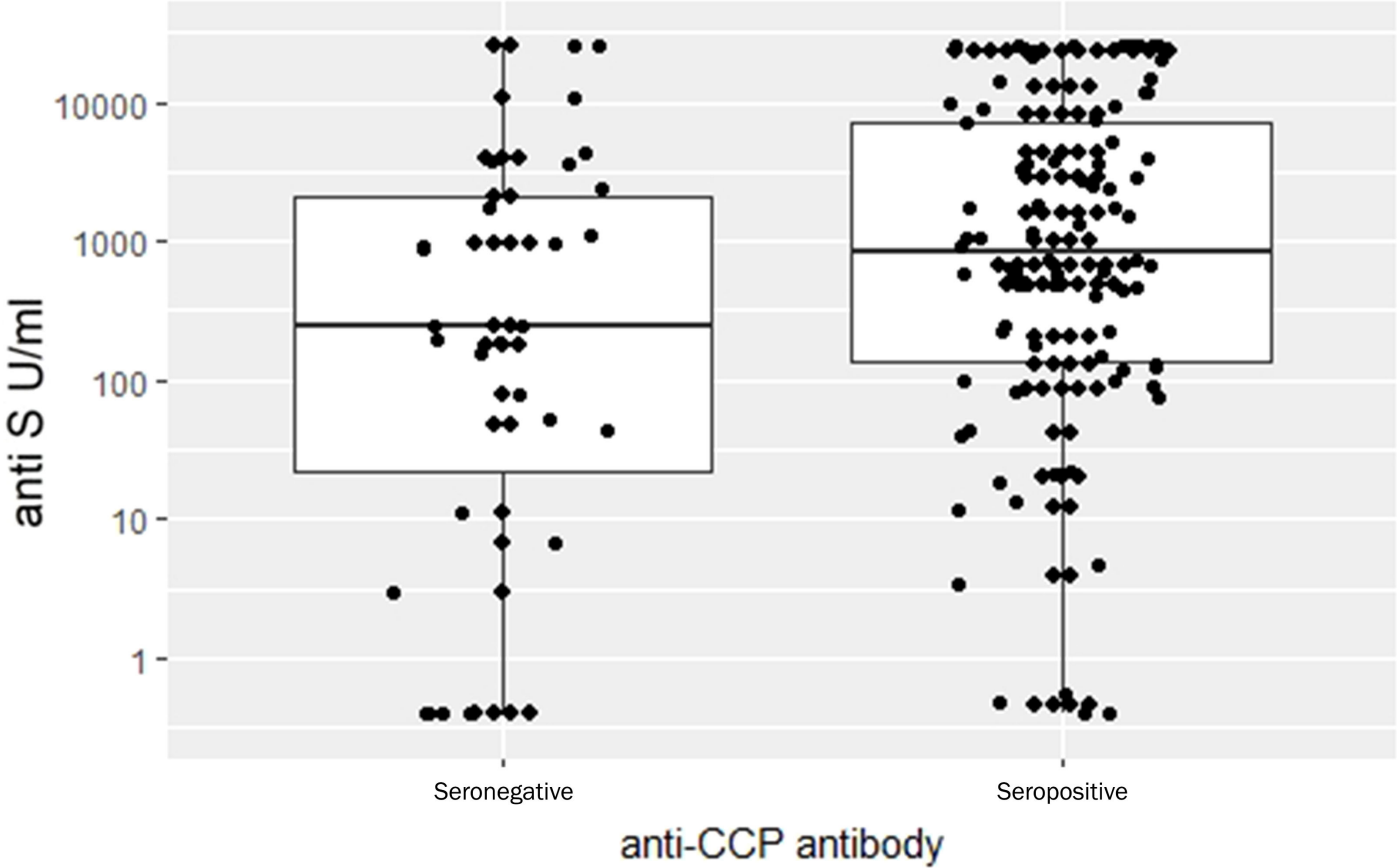
Comparison anti-S Ig levels between RA anti-CCP seronegative and seropositive patients. Observed data. Detailed statistical analysis showed that the difference between the groups is highly significant (p= 0.00165).

The regression model predicted that in presence of aCCP, anti-S levels increased by 6.28 times, a highly significant result (p= 0.00165). Patients with RF present were expected to have 3.33 times higher anti-S levels than patients without it (p = 0.047). When both factors were entered into the regression equation only the effect of aCCP remained significant (p = 0.01515) and the multiplying effect practically remained the same (6.06). We interpret this result as sign that aCCP is more directly linked to the humoral response than RF. Neither CRP nor DAS28-CRP had any significant effect on the anti-S levels and none of these covariates had any influence on T-cell mediated immune response markers. **Figure 6** illustrates in anti-S immune response between aCCP negative and positive patients. Note that these are the observed data while the statistical conclusion is based upon the covariate adjusted data. This distinction is important because many potential explanations for the observed phenomenon shown in **Figure 6** can be excluded. For example, it can be excluded that anti-S level difference between aCCP positive and negative patients is due to treatment unbalance between the two groups.

## Discussion

In this study conducted during the COVID-19 pandemic, we demonstrate several major factors modifying the COVID-19 infection and vaccination induced humoral and cellular immune response in patients with RA. COVID-specific humoral immune response was detected in the majority of RA patients, with a seropositivity rate of 94%; interestingly aCCP positive patients had substantially higher anti-S levels than aCCP negative patients. Our present data confirm and extend previous observations regarding the effect of targeted therapies on the COVID specific immune response in RA; the levels of anti-S antibodies were significantly lower among patients treated with TNF-α or IL-6-inhibitor than that of the healthy donors’ (19–22). Furthermore, in the case of rituximab-treated RA patients the COVID-specific humoral and cellular immune responses were both attenuated (23).

Our present data support findings suggesting that COVID-specific humoral and cellular immune responses wane with age (4%/year and 2%/year decrease, respectively) and diminish over time (25%/month and 6%/month, respectively) (24). Previous clinical studies proved the immunogenicity and safety of SARS-CoV-2 vaccination in adult patients with autoimmune inflammatory rheumatic diseases (22, 25). Our study population was heterogeneous according to the types of COVID vaccinations. We studied the effect of five types of vaccines against COVID-19. We found that mRNA-1273, BNT162b2 and ChAdOx1s vaccinations potently increased the anti-S antibody levels, while based on our results, Gam-COVID vaccine and BBIBP-CorV vaccine had no significant impact on the antibody response. Recently, it has been published, that individuals who received Gam-COVID or mRNA-1273 vaccines had higher COVID-specific T-cell response compared to the inactivated viral vaccine (26).

Our results provide detailed information considering the effect of various immunosuppressive therapies on COVID-specific immune responses months after COVID infection or the last vaccine administration (median time in months: 3.67 (2.03, 5.50)). Based on recent studies, rituximab treatment was the strongest predictor of failure to seroconvert (22, 27–29); this is consistent with our present data: we detected 97% (p=0.002) lower COVID-specific humoral immune responses in those who were treated with anti-CD20 therapy. It was also reported that MTX can induce a significant reduction of antibody levels compared to healthy controls (30), although at a lesser extent than with anti-CD20 treatment (25). By contrast, based on our current results, MTX had no significant effect on the anti-S antibody levels (p=0.087) compared to the same patient not treated with MTX therapy. This could be explained by the short-term effect of MTX on the COVID-specific humoral immune response. In our study, we examined the effect of MTX on anti-S antibody levels in patients diagnosed with RA, whereas in other studies patients with other systemic autoimmune diseases were also included (30). Previous data regarding the impact of targeted therapies such as anti-TNF, anti-IL-6 biologicals and JAK inhibitors on COVID-specific immune response are contradictory. According to our present data, IL-6 inhibitor tocilizumab had a negative impact on the COVID-specific humoral immune response (p=0.049), similarly to other previously published observations (20, 21). This is likely due to the effect of IL-6 in controlling the maturation, expansion and survival of B-cells (31). In contrast with other studies (21, 32), we found that TNF-α inhibitors significantly decreased the anti-S antibody levels (by 80%, p <0.001) in the RA group. The disparate effect of TNF-α inhibitors could be explained by the longer sampling interval and the use of various types of vaccines in our research. Furthermore, similarly to our present data, it has been recently shown that the low dose CCS treatment did not affect anti-SARS-CoV-2 responses (32).

Based on our findings, compared with healthy controls (87%), a lower proportion of RA patients had detectable COVID-specific T-cell response (52%), similarly to other previous reports regarding patients with immune-mediated inflammatory diseases (20, 33). In patients who received anti-CD20 treatment, we observed 65% decreased COVID-specific T cell-mediated immune response compared to the controls (p=0.055). By contrast, based on a recent study, patients treated with rituximab were able to mount robust T-cell responses to mRNA COVID-19 vaccines, despite the decreased anti-S antibody levels (34). According to the results of a study, where the COVID-specific T-cell response was studied two weeks after the second BNT162b2 vaccination dose, RA patients receiving TNF-α inhibitors and IL-6 inhibitor had substantially impaired COVID-specific T-cell response than that of the controls’ (21). In contrast to the previous study, our study showed that some targeted therapies (e.g., TNF-α inhibitors, IL-6 inhibitor and JAK inhibitors) had no significant impact on COVID-specific cellular immune response; this might be explained that we assessed the specific T-cell response during substantially longer time (median time in months: 3.67 (2.03, 5.50)).

This is an observational study with its inherent limitations. One of the limitations is that Group factor (healthy/RA) was confounded with the immunosuppressive treatment and patients with RA were significantly older compared to the healthy control group. Nevertheless, according to our present data/results, the estimated effect of the commonly used biologicals is robust regardless of these confounding factors. First, we got essentially the same effects excluding data of the healthy control groups. Second, inclusion of the group factor did not change the conclusions. Still, it is a question that the disease itself has an additional effect because the estimated effects are not negligible. Further subgroup analysis was not possible due to the relatively low number of patients.

Further studies on the clinical relevance of changes in COVID-specific immune response over time are necessary to understand the long-term effects of the main influencing factors. Our results suggest that, repeat vaccination boost strategies should be considered in RA to provide sustained and effective COVID specific humoral and cellular immune response. In addition, future studies are needed to investigate heterologous vaccination scheme.

## Data availability statement

The original contributions presented in the study are included in the article/**Supplementary Material**. Further inquiries can be directed to the corresponding author.

## Ethics statement

The studies involving human participants were reviewed and approved by National Public Health Center, Hungary. IV/2021-1/2021/EKU. The patients/participants provided their written informed consent to participate in this study.

## Author contributions

DN performed design of the study, data collection, first draft of the manuscript, and data interpretation; LT performed statistical analysis; HV, ZU, DB, ZS, and BR performed data interpretation; BM and GN performed design of the study, manuscript preparation and data analysis. All authors gave their final approval to the manuscript.

## Funding

This project was financed by the National Research, Development and Innovation Office Fund in Hungary (2020-2.1.1-ED-2022-00198), the Thematic Excellence Programme (Tématerületi Kiválósági Program, TKP2021-EGA-29) of the Ministry for Innovation and Technology in Hungary, OTKA Grant (K 131479). This study was supported by the National Research, Development and Innovation Office of Hungary under the Investement in the Future funding scheme (2020-1.1.6-JÖVŐ-2021-00013) and by the grant of European Union RRF-2.3.1-21-2022-00003. TKP2021-EGA-23 has been implemented with the support provided by the Ministry of Innovation and Technology of Hungary from the National Research, Development and Innovation Fund, financed under the TKP2021-EGA funding scheme.

## Acknowledgments

We thank Ilona Dargai for her contribution to our work. We acknowledge the laboratory support of Zsolt Pósa.

## Conflict of interest

The authors declare that the research was conducted in the absence of any commercial or financial relationships that could be construed as a potential conflict of interest.

## Publisher’s note

All claims expressed in this article are solely those of the authors and do not necessarily represent those of their affiliated organizations, or those of the publisher, the editors and the reviewers. Any product that may be evaluated in this article, or claim that may be made by its manufacturer, is not guaranteed or endorsed by the publisher.
