# Supplementary material for: Factors influencing the SARS-CoV-2 infection and vaccination induced immune response in rheumatoid arthritis
Source: Front Immunol. 2022 Oct 12;13:960001. doi: 10.3389/fimmu.2022.960001 (PMC9596981; doi:10.3389/fimmu.2022.960001)
Supplement: Supplementary file 1 [file Table_1.docx]

Supplementary Material

# Supplementary Tables

**Supplementary Table 1.** The dependence of logtransformed anti-S antibodies on demographic parameters was examined by linear regression. The table shows the exponetiated regression coefficients and their 95% confidence interval. Both cohorts (healthy and RA patients).

| Characteristic | exp (Beta) | 95% CI^1^ | p-value |
| --- | --- | --- | --- |
| Group | 0.42 | 0.09, 1.94 | 0.3 |
| Sampling Interval | 0.76 | 0.66, 0.87 | <0.001 |
| Vaccination |  |  |  |
| ChAdOx1s | 24.2 | 1.51, 390 | 0.026 |
| BNT162b2 | 41.4 | 6.29, 272 | <0.001 |
| Gam-COVID | 2.90 | 0.17, 49.5 | 0.5 |
| mRNA-1273 | 89.0 | 10.7, 739 | <0.001 |
| BBIBP-CorV | 0.25 | 0.01, 4.11 | 0.3 |
| Gender | 1.17 | 0.39, 3.49 | 0.8 |
| Age | 0.96 | 0.93, 0.99 | 0.014 |
| Targeted therapies |  |  |  |
| anti-CD20 | 0.02 | 0.00, 0.28 | 0.003 |
| IL-6 inhibitor | 0.35 | 0.08, 1.42 | 0.14 |
| JAK inhibitor | 2.58 | 0.28, 23.8 | 0.4 |
| TNF-α inhibitor | 0.27 | 0.10, 0.76 | 0.014 |
| MTX | 2.21 | 0.90, 5.44 | 0.087 |
| CCS | 0.60 | 0.20, 1.74 | 0.3 |
| ^1^CI = Confidence Interval | | | |

**Supplementary Table 2.** The effect of RA on anti-S antibodies. The results were also examined by fitting a regression model to the data of RA patients only. The regression parameter estimates in the table are essentially the same as those obtained in the complete data set.

| Characteristic | exp (Beta) | 95% CI^1^ | p-value |
| --- | --- | --- | --- |
| Sampling Interval | 0.77 | 0.64, 0.94 | 0.012 |
| Vaccination |  |  |  |
| ChAdOx1s | 66.0 | 0.92, 4,762 | 0.058 |
| mRNA-1273 | 173 | 6.61, 4,509 | 0.003 |
| BNT162b2 | 82.6 | 3.45, 1,978 | 0.008 |
| BBIBP-CorV | 0.56 | 0.01, 28.3 | 0.8 |
| Gam-COVID | 5.09 | 0.01, 1,922 | 0.6 |
| Gender | 1.56 | 0.28, 8.68 | 0.6 |
| Age | 0.95 | 0.92, 0.99 | 0.021 |
| Targeted therapies |  |  |  |
| anti-CD20 | 0.02 | 0.00, 0.36 | 0.008 |
| IL-6 inhibitor | 0.37 | 0.08, 1.77 | 0.2 |
| JAK inhibitor | 3.18 | 0.25, 39.8 | 0.4 |
| TNF-α inhibitor | 0.27 | 0.09, 0.86 | 0.028 |
| MTX | 2.36 | 0.86, 6.46 | 0.10 |
| CCS | 0.64 | 0.19, 2.15 | 0.5 |
| ^1^CI = Confidence Interval | | | |

**Supplementary Table 3.** The dependence of logtransformed whole COVID virus-specific CD4+ and CD8+ T-cell response on the demographic parameters. Both cohorts.

| Characteristic | exp (Beta) | 95% CI^1^ | p-value |
| --- | --- | --- | --- |
| Group | 0.50 | 0.25, 1.03 | 0.061 |
| Sampling Interval | 0.94 | 0.88, 1.01 | 0.073 |
| Vaccination |  |  |  |
| ChAdOx1s | 1.30 | 0.27, 6.33 | 0.7 |
| BNT162b2 | 0.68 | 0.20, 2.28 | 0.5 |
| Gam-COVID | 0.53 | 0.10, 2.80 | 0.5 |
| mRNA-1273 | 1.43 | 0.41, 4.98 | 0.6 |
| BBIBP-CorV | 0.36 | 0.08, 1.61 | 0.2 |
| Gender | 1.12 | 0.68, 1.87 | 0.7 |
| Age | 0.99 | 0.97, 1.00 | 0.053 |
| Targeted therapies |  |  |  |
| anti-CD20 | 0.35 | 0.12, 1.00 | 0.052 |
| IL-6 inhibitor | 1.12 | 0.60, 2.09 | 0.7 |
| JAK inhibitor | 1.14 | 0.42, 3.08 | 0.8 |
| TNF-α inhibitor | 1.07 | 0.68, 1.67 | 0.8 |
| MTX | 1.20 | 0.81, 1.79 | 0.4 |
| CCS | 0.66 | 0.41, 1.06 | 0.091 |
| ^1^CI = Confidence Interval | | | |

**Supplementary Table 4.** The effect of RA on whole COVID virus-specific CD4+ and CD8+ T-cell response. The results were also examined by fitting a regression model to the data of RA patients only. The regression parameter estimates thus obtained are essentially the same as those obtained in the complete data set.

| Characteristic | exp(Beta) | 95% CI^1^ | p-value |
| --- | --- | --- | --- |
| Sampling Interval | 0.92 | 0.85, 1.00 | 0.051 |
| Vaccination |  |  |  |
| ChAdOx1s | 1.40 | 0.25, 7.92 | 0.7 |
| mRNA-1273 | 1.34 | 0.36, 5.03 | 0.7 |
| BNT162b2 | 0.62 | 0.17, 2.25 | 0.5 |
| BBIBP-CorV | 0.35 | 0.07, 1.72 | 0.2 |
| Gam-COVID | 0.36 | 0.03, 4.05 | 0.4 |
| Gender | 1.56 | 0.78, 3.13 | 0.2 |
| Age | 0.98 | 0.97, 1.00 | 0.046 |
| Biological |  |  |  |
| anti-CD20 | 0.32 | 0.11, 0.96 | 0.044 |
| IL-6 inhibitor | 1.15 | 0.61, 2.18 | 0.7 |
| JAK inhibitor | 1.14 | 0.41, 3.18 | 0.8 |
| TNF-α inhibitor | 1.05 | 0.66, 1.66 | 0.9 |
| MTX | 1.21 | 0.81, 1.83 | 0.4 |
| CCS | 0.72 | 0.44, 1.18 | 0.2 |
| ^1^CI = Confidence Interval | | | |
